# Polyclonal Pulmonary Tuberculosis Infections and Risk for Multidrug Resistance, Lima, Peru

**DOI:** 10.3201/eid2311.170077

**Published:** 2017-11

**Authors:** Ruvandhi R. Nathavitharana, Cynthia X. Shi, Leonid Chindelevitch, Roger Calderon, Zibiao Zhang, Jerome T. Galea, Carmen Contreras, Rosa Yataco, Leonid Lecca, Mercedes C. Becerra, Megan B. Murray, Ted Cohen

**Affiliations:** Beth Israel Deaconess Medical Center, Boston, Massachusetts, USA, and Imperial College London, London, UK (R.R. Nathavitharana);; Yale School of Public Health, New Haven, Connecticut, USA (C.X. Shi, T. Cohen);; Simon Fraser University, Burnaby, British Columbia, Canada (L. Chindelevitch);; Socios En Salud Sucursal Peru, Lima, Peru (R. Calderon, J.T. Galea, C. Contreras, R. Yataco, L. Lecca);; Harvard Medical School, Boston (Z. Zhang, J.T. Galea, L. Lecca, M.C. Becerra, M.B. Murray)

**Keywords:** tuberculosis, TB, strain diversity, multidrug-resistant TB, polyclonal infection, Mycobacterium tuberculosis, bacteria, mycobacterial interspersed repetitive units–variable number of tandem repeats, MIRU-VNTR, classifier of tandem repeats, ClassTR, Peru, drug susceptibility testing, antimicrobial resistance, tuberculosis and other mycobacteria

## Abstract

Because within-host *Mycobacterium tuberculosis* diversity complicates diagnosis and treatment of tuberculosis (TB), we measured diversity prevalence and associated factors among 3,098 pulmonary TB patients in Lima, Peru. The 161 patients with polyclonal infection were more likely than the 115 with clonal or the 2,822 with simple infections to have multidrug-resistant TB.

Within-host heterogeneity of *Mycobacterium tuberculosis* infection is increasingly recognized as an obstacle for the accurate diagnosis (*1*) and effective treatment (*2*) of tuberculosis (TB) and may complicate the control of TB in communities (*3*). Within-host heterogeneity may arise through 2 mechanisms: 1) by reinfection or simultaneous infection with multiple strains, which results in a polyclonal (mixed) infection, or 2) by accumulation of mutations, which results in clonal heterogeneity (*4*). The treatment challenge posed by within-host heterogeneity has been most clearly demonstrated for infections with drug-susceptible and drug-resistant variants (*5*). The relatively high prevalence of multidrug-resistant (MDR) TB in Peru (≈6% among new case-patients and 21% among retreatment case-patients) (*6*) places increased stress on the healthcare system.

Our main objectives were to estimate the prevalence of within-host *M. tuberculosis* heterogeneity at the time of treatment initiation in a large cohort of pulmonary TB patients in Peru and to determine if factors measurable at the baseline visit were associated with complex infections (*7*). To determine whether our insights were sensitive to the method used for distinguishing between classes of heterogeneous infections, we used a newly described method (classifier of tandem repeats [ClassTR]) (*8*), which uses 24-loci mycobacterial interspersed repetitive units–variable number of tandem repeats (MIRU-VNTR) data to distinguish polyclonal and clonal infections, and we compared these findings with an analysis based on the standard threshold-based approach (*9*). 

## The Study

During September 2009–August 2012, we attempted to enroll all adults (>15 years of age) with a diagnosis of incident pulmonary TB from 106 healthcare centers in Lima, Peru; details of the study design have been reported previously (*7*). We recorded baseline data on demographics, medical history, and results of drug susceptibility testing (DST) for rifampin, isoniazid, streptomycin, ethambutol, and pyrazinamide. We restricted our analysis to pretreatment samples and data from participants with culture-positive TB from whom sufficient mycobacterial DNA could be successfully obtained from the baseline sample to perform MIRU-VNTR typing.

All enrolled index case-patients and household contacts evaluated for active TB were assessed by sputum smear microscopy with Ziehl-Neelsen staining and culture on solid Lowenstein-Jensen medium. Initial DST was performed by using the proportion method on Lowenstein-Jensen medium; second-line DST was performed by using the proportion method on Middlebrook 7H11 agar. We shipped 100 μL of the lysate from suspensions of mycobacterial colonies harvested from Lowenstein-Jensen slants to Genoscreen (Institute Pasteur, Lille, France) for 24-loci MIRU-VNTR typing.

The standard threshold approach for classifying complex infections by using MIRU-VNTR data classifies patterns with >1 band (i.e., repeat copy number) at a single locus as clonal infections and patterns with >1 band at multiple loci as polyclonal infections (*9*). To better distinguish between clonal and polyclonal infections, we used an alternative method called ClassTR, which leverages additional information about differences in loci copy numbers and from other strains present in the population (*8*). In simulation studies, ClassTR more accurately distinguished between these 2 mechanisms of within-host diversity than did the threshold approach (*8*).

To understand whether our findings were robust to the classification approach used, we adopted ClassTR for our main analysis, but we also repeated all analyses with the threshold approach. We used univariable and multivariable multinomial logistic regression to identify baseline factors independently associated with having a clonal or a polyclonal infection, setting simple infection as the referent. Analysis was limited to complete cases. Co-linearity was assessed by calculating variance inflation factors, and p<0.05 was considered statistically significant. Co-linear variables were removed to produce the final multivariable model. Statistical analyses were conducted in R version 3.3 (http://www.R-project.org). Research ethics committees in Peru and Boston approved the study.

We analyzed results for 3,098 participants. Most participants were <35 years of age (64.8%) and male (61.9%); 108 (3.5%) were known to be HIV infected. Nearly a fifth (18.8%) of participants reported a prior history of TB, and 78 (2.5%) reported having received a course of isoniazid chemoprophylaxis. A total of 375 (12.1%) participants had MDR-TB, 288 (9.3%) had isoniazid or rifampin monoresistance, and 357 (11.5%) had other resistance patterns (predominantly streptomycin resistance). A total of 2,822 (91.1%) participants had simple infections (i.e., no evidence of within-host heterogeneity by MIRU-VNTR), and the remaining 276 (8.9%) had evidence of within-host heterogeneity. Using ClassTR, we classified 161 (5.2%) infections as polyclonal and 115 (3.7%) as clonal (Table 1). 

**Table 1:** *Mycobacterium tuberculosis* resistance patterns among patients with pulmonary TB, Lima, Peru, September 2009–August 2012*

Resistance†	Simple, no. (%)	Clonal, no. (%)	Polyclonal, no. (%)
Pansensitive	1,917 (67.9)	73 (63.5)	88 (54.7)
INH or RIF resistance	260 (9.2)	11 (9.6)	17 (10.6)
Multidrug	333 (11.8)	15 (13.0)	27 (16.8)
Other	312 (11.1)	16 (13.9)	29 (18.0)
Total	2,822	115	161

*Mycobacterium tuberculosis strain type determined by classifier of tandem repeats. INH, isoniazid; RIF, rifampin; TB, tuberculosis. †Drug susceptibility testing was performed for RIF, INH, streptomycin, ethambutol, and pyrazinamide.

Multivariable multinomial logistic regression results associated polyclonal infection with multidrug resistance (adjusted odds ratio 1.66, 95% CI 1.05–2.62; p = 0.03) and other drug resistance (adjusted odds ratio 1.97, 95% CI 1.27–3.06; p = 0.002) (Table 2). No factors were significantly associated with clonal infection in either univariable or multivariable analysis. These associations were largely preserved when we repeated the analysis by using the threshold classification approach (Technical Appendix Tables 1, 2). 

**Table 2:** Factors associated with clonal and polyclonal *Mycobacterium tuberculosis* infection among patients with pulmonary TB, Lima, Peru, September 2009–August 2012*

Characteristic	Clonal aOR (95% CI), n = 115	p value	Polyclonal aOR (95% CI), n = 161	p value
Age, y				
15–24	Referent		Referent	
25–34	1.21 (0.75–1.96)	0.44	1.40 (0.93–2.11)	0.11
35–44	1.16 (0.64–2.11)	0.62	1.42 (0.87–2.31)	0.16
>45	1.33 (0.79–2.23)	0.29	1.22 (0.77–1.95)	0.40
Male sex	0.93 (0.63–1.38)	0.72	1.10 (0.78–1.55)	0.59
Previous TB	0.82 (0.48–1.37)	0.45	1.27 (0.86–1.86)	0.23
Previous INH receipt	1.51 (0.54–4.27)	0.44	1.84 (0.82–4.15)	0.14
HIV infection	0.99 (0.35–2.78)	0.98	1.12 (0.50–2.49)	0.79
≥1 chronic disease	0.90 (0.55–1.46)	0.66	0.97 (0.64–1.46)	0.88
Hospitalized	1.01 (0.58–1.75)	0.97	0.98 (0.61–1.57)	0.93
Resistance pattern				
Pansensitive	Referent		Referent	
INH or RIF resistance	1.11 (0.58–2.13)	0.76	1.38 (0.81–2.37)	0.24
Multidrug resistance	1.24 (0.70–2.22)	0.46	1.66 (1.05–2.62)	0.03
Other	1.34 (0.77–2.33)	0.31	1.97 (1.27–3.06)	0.002

*Results of multivariable regression analysis using classifier of tandem repeats method. aOR, adjusted odds ratio; INH, isoniazid; RIF, rifampin; TB, tuberculosis.

## Conclusions

Among a large cohort of pulmonary TB patients in Lima, Peru, we found evidence of within-host *M. tuberculosis* diversity at the time of treatment initiation in ≈9%. The ClassTR approach for classification based on MIRU-VNTR typing indicated that 5.2% of patients had polyclonal infections and 3.7% had clonal infections.

Polyclonal infections were positively associated with multidrug resistance and other drug-resistance patterns. When we used a 2-sided exact binomial test to test the hypothesis that the risk for multidrug resistance among participants with polyclonal infection (using the observed fraction 27/161) differed from that expected with infection by 2 randomly selected strains, calculated as 1 – (1 – 348/2,937)^2^, we obtained a p value of 0.11. This p value suggests that, at least in this setting, the association between polyclonal infection and multidrug resistance cannot be attributed to more than the increased risk that would accrue from multiple exposures.

A review of the literature on factors associated with within-host diversity revealed substantial variability between settings. Studies from Botswana and Taiwan found a higher prevalence of polyclonal infection among patients with MDR-TB (*10*,*11*); however, studies from Vietnam and KwaZulu-Natal (South Africa) did not find this association (*12**,**13*). It is possible that this association may be modified in the presence of HIV coinfection or that the ability to identify such an association is easier in areas where the prevalence of multidrug resistance is higher.

The main strengths of this study relate to the large prospective cohort of pulmonary TB patients evaluated in a study area with a population of 3.3 million persons. However, 30% of enrolled participants did not have culture-confirmed TB, precluding MIRU-VNTR analysis on all participants. Use of the MIRU-VNTR assay on cultured specimens to detect within-host heterogeneity was motivated by practical considerations. Because MIRU-VNTR typing is unable to identify all minority variants, and some diversity may be lost during culture (*14*), our categorization of infections into simple, clonal, and polyclonal may be subject to misclassification, which would be differential (i.e., complex infections are more likely to be misclassified as simple than the reverse) and could lead to bias. The use of a high number of MIRU-VNTR loci also reduces the likelihood of homoplasy. Furthermore, although the biological clock of the MIRU-VNTR marker seems to be relatively stable (recently estimated MIRU-VNTR mutation rate for TB is 2.70 × 10^–3^ mutations/locus/year [*15*]), changes accruing in the marker could lead to misclassification of clonal strains as polyclonal strains; we used the ClassTR method in an attempt to minimize such misclassification. 

We found complex infections attributable to multiple infection events to be associated with increased risk for MDR TB. This finding further emphasizes the value of efforts to mitigate the transmission of MDR TB. 

Technical Appendix Factors associated with clonal and polyclonal multidrug-resistant tuberculosis infection determined by using the classifier of tandem repeats approach; resistance patterns according to *Mycobacterium tuberculosis* strain type determined by threshold classification approach.
