# Supplementary material for: Polyclonal Pulmonary Tuberculosis Infections and Risk for Multidrug Resistance, Lima, Peru
Source: Emerg Infect Dis. 2017 Nov;23(11):1887–90. doi: 10.3201/eid2311.170077 (PMC5652442; doi:10.3201/eid2311.170077)
Supplement: Technical Appendix — Factors associated with clonal and polyclonal multidrug-resistant tuberculosis infection determined by using the classifier of tandem repeats approach; resistance patterns according to Mycobacterium tuberculosis strain type determined by threshold classification approach. [file 17-0077-Techapp-s1.pdf]

Article DOI: https://doi.org/10.3201/eid2311.170077

Polyclonal Pulmonary Tuberculosis Infections and Risk for Multidrug Resistance, Lima, Peru

Technical Appendix

Technical Appendix Table 1. Results of univariable regression analysis of factors associated with clonal and polyclonal MTB infection using ClassTR classification approach

Characteristic	Clonal OR N = 115	95% CI		p value	Polyclonal OR N = 161	95% CI		p value
Age, y								
15–24	Ref				Ref			
25–-34	1.18	0.73	1.90	0.50	1.44	0.96	2.15	0.08
35–44	1.11	0.62	2.00	0.72	1.48	0.92	2.39	0.11
≥45	1.23	0.75	2.03	0.41	1.23	0.79	1.91	0.37
Male sex	0.93	0.63	1.36	0.70	1.13	0.81	1.57	0.48
Previous TB	0.87	0.53	1.44	0.59	1.45	1.01	2.10	0.05
Previous INH	1.48	0.53	4.14	0.45	1.87	0.84	4.14	0.12
HIV-Positive	1.01	0.37	2.80	0.98	1.28	0.58	2.80	0.54
≥1 Chronic disease	0.97	0.61	1.53	0.89	0.98	0.67	1.45	0.93
Hospitalized	1.03	0.60	1.76	0.92	1.01	0.64	1.60	0.97
Resistance pattern								
Pan-sensitive	Ref				Ref			
INH or RIF resistance	1.11	0.58	2.12	0.75	1.42	0.83	2.43	0.20
MDR	1.18	0.67	2.09	0.56	1.77	1.13	2.76	0.01
Other DR	1.35	0.77	2.34	0.29	2.03	1.31	3.13	0.002

Technical Appendix Table 2. Resistance Patterns according to MTB Strain Type determined by threshold classification approach

Resistance*	Simple N (%)	Clonal N (%)	Polyclonal N (%)
Pan-Sensitive	1917 (67.9)	112 (68.7)	49 (43.4)
INH or RIF resistance	260 (9.2)	17 (10.4)	11 (9.7)
MDR	333 (11.8)	11 (6.7)	31 (27.4)
Other	312 (11.1)	23 (14.1)	22 (19.5)
Total	2822	163	113

*Drug susceptibility testing was performed for the following drugs: rifampicin, isoniazid, streptomycin, ethambutol, and pyrazinamide.

Technical Appendix Table 3. Results of multivariable regression analysis of factors associated with clonal and polyclonal MTB infection using threshold classification approach

Characteristic	Clonal aOR N = 115	95% CI		p value	Polyclonal aOR N = 161	95% CI		p value
Age, y								
15–24	Ref				Ref			
25–34	1.37	0.91	2.07	0.13	1.22	0.75	1.97	0.42
35–44	1.53	0.94	2.48	0.09	1.04	0.56	1.91	0.91
≥45	1.36	0.87	2.12	0.18	1.16	0.67	2.02	0.60
Male sex	0.98	0.70	1.36	0.88	1.13	0.75	1.71	0.56
Previous TB	0.72	0.46	1.15	0.17	1.63	1.06	2.50	0.03
Previous INH	1.13	0.40	3.18	0.81	2.48	1.08	5.72	0.03
HIV-Positive	0.67	0.24	1.86	0.44	1.67	0.73	3.81	0.22
≥1 Chronic disease	1.04	0.70	1.55	0.85	0.78	0.46	1.31	0.35
Hospitalized	1.09	0.69	1.72	0.71	0.85	0.47	1.52	0.58
Resistance pattern								
Pan-sensitive	Ref				Ref			
INH or RIF resistance	1.11	0.65	1.88	0.71	1.59	0.82	3.11	0.17
MDR	0.60	0.32	1.14	0.12	3.25	2.01	5.26	<0.001
Other DR	1.25	0.78	1.99	0.35	2.70	1.61	4.55	<0.001

Technical Appendix Table 4. Results of univariable regression analysis of factors associated with clonal and polyclonal MTB infection using threshold classification approach

Characteristic	Clonal OR N = 115	95% CI		p value	Polyclonal OR N = 161	95% CI		p value
Age, y								
15–24	Ref				Ref			
25–-34	1.32	0.88	1.99	0.18	1.32	0.83	2.11	0.24
35–-44	1.45	0.90	2.34	0.13	1.14	0.63	2.06	0.66
≥45	1.34	0.88	2.06	0.18	1.08	0.64	1.82	0.78
Male sex	0.93	0.68	1.29	0.67	1.22	0.82	1.82	0.33
Previous TB	0.72	0.46	1.13	0.16	2.06	1.37	3.09	0.001
Previous INH	1.03	0.37	2.87	0.95	2.72	1.22	6.06	0.02
HIV-Positive	0.71	0.26	1.95	0.50	1.86	0.84	4.09	0.13
≥1 Chronic disease	1.12	0.77	1.62	0.56	0.79	0.48	1.29	0.35
Hospitalized	1.10	0.70	1.72	0.68	0.90	0.51	1.59	0.72
Resistance pattern								
Pan-sensitive	Ref				Ref			
INH or RIF resistance	1.12	0.66	1.89	0.68	1.66	0.85	3.22	0.14
MDR	0.57	0.30	1.06	0.08	3.64	2.29	5.80	<0.001
Other DR	1.26	0.79	2.01	0.33	2.76	1.65	4.63	<0.001
